# Tripartite interaction among teachers, parents and young children in Chinese parent–child program

**DOI:** 10.3389/fpsyg.2025.1602763

**Published:** 2025-08-19

**Authors:** Jiali Kang, Kamariah Abu Bakar, Suziyani Mohamed

**Affiliations:** ^1^Faculty of Education, Universiti Kebangsaan Malaysia, Bangi, Malaysia; ^2^Changshu Institute of Technology, Changshu, Jiangsu, China

**Keywords:** parent–child program, tripartite interaction, early childhood education, event sampling, China, teacher-parent-child relationship

## Abstract

**Introduction:**

Tripartite interaction among teachers, parents, and young children is a cornerstone of early childhood education, especially in China’s parent-child programs designed to support children’s development and enhance parental engagement. Despite their intentions, these interactions often follow rigid patterns, limit children’s agency, and involve minimal communication between adults. This research investigates the dynamics and challenges of such tripartite interactions within Chinese preschools.

**Methods:**

A qualitative approach was adopted using purposive sampling in two preschools in Nanjing, China. Participants included 29 children (Mage = 30 months), their parents, and four teachers, engaged in 28 structured parent-child activities. Data collection comprised 1,130 observed tripartite interaction events using event sampling and 23 semi-structured and impromptu interviews with teachers and parents. The data were coded and analyzed in NVivo12 through open, axial, and selective coding to extract interaction types and thematic patterns.

**Results:**

Findings show that adult-initiated interactions (teacher-child-parent and parent-child-teacher types) accounted for 86% of events, primarily reflecting an Initiation-Response-Evaluation (IRE) pattern led by adults. Children’s attempts to initiate were least accepted, indicating a suppression of their subjectivity. Six themes emerged: education and instruction, encouragement and communication, assistance and substitution, guidance through concerns, mutual understanding, and children’s self-expression. A new tripartite interaction model was developed.

**Discussion:**

This research underscores the need for restructuring Chinese parent-child programs by promoting balanced goals, recognizing each party’s role, and cultivating respectful, child-inclusive dialogue to support holistic development.

## 1 Research background

Interaction is one of the essential indicators for evaluating the quality of early education (Pianta et al., 2005; McGuire et al., 2016; Karen et al., 2004). The Strong Start Report published by the OECD (2017) Education Committee recognizes that children’s daily interactions in Early Childhood Education (ECE) settings reflect the quality of ECE they experience. Numerous studies have indicated that high-quality interactions facilitate the enhancement of young children’s capabilities, inspire their eagerness to learn, and contribute to the advancement of their cognitive skills, including language, mathematics, and scientific exploration (Rüdisüli et al., 2024; Li and Yu, 2023; Karen et al., 2004; Smidt and Lehrl, 2018; Lehrl and Smidt, 2017). A systematic review underscored the vital significance of early interactions, noting that variations in interaction quality can significantly influence developmental outcomes (Howard et al., 2024). Observational research demonstrates that both the quality and frequency of interactions during daily activities, such as free play, are essential for promoting development (Frick and Lehnerer, 2023).

Research indicates that for children aged 2–3 years, the quality of interactions in childcare environments is positively associated with working memory and reduced disruptive behavior (Gerlind et al., 2022). Conversely, some studies suggest that not all types of interactions yield beneficial outcomes. For example, certain teaching methodologies adversely affect children’s play (Rüdisüli et al., 2024). This underscores the intricate nature of interaction quality in early childhood environments.

Parent–child programs in China are a set of structured play activities designed by teachers to support the development of young children ages 0–3. It is aimed at enhancing parental involvement in children’s education. Parental engagement helps children to establish secure attachment relationships (Pleck, 2007) and engage in increasingly complex social interactions (Bronfenbrenner, 1986). A research in Zurich demonstrated that parents who participated in Parent–Child Interaction Training reported significant improvements in their children’s behavior, independent of initial problem severity (Zulauf-Logoz and Zelger, 2022). Research in Italy highlighted that parenting courses increased parental awareness of educational activities, leading to more time spent reading and engaging with children (Boca et al., 2022). Similarly, a research in Malaysia found that mothers who completed a parent training program observed significant improvements in their children’s behavior (Masiran et al., 2023).

Parent–child programs are activities in which teachers, parents, and young children construct experiences through the interaction of multiple factors. Tripartite interaction is a process of interplay and mutual influence on the same subject, initiated by one party through verbal and non-verbal (e.g., tone of voice, gaze, gestures, etc.) interactions at the same time and in the same domain, with the participation of all three parties. Collaborative pedagogical interactions among parents, children, and teachers are essential for holistic child development. Positive relationships, teamwork, and innovative methods enhance intellectual, emotional, and social growth in preschoolers (Levinthal et al., 2021).

Two models are involved in the examination of interpersonal interaction: the structural paradigm and the process paradigm. The structural paradigm prioritizes the examination of group structure and characteristics in the analysis of individual interactions, employing operational and quantitative research methodologies to investigate interaction characteristics (Ye and Pang, 2009). The process paradigm contends that while interpersonal interactions occur within groups possessing stable structural characteristics, individual interactions are predominantly shaped by the participants’ perceptions and interpretations of the context and each other’s behaviors, highlighting the variability of interactions (Westermann and Sibilis, 2022). The process paradigm asserts that researchers must thoroughly examine the actual interaction, analyzing its initiation, theme, flow, and outcome concerning the specific and intricate behaviors and content exhibited by the subject during the interaction (Cai, 2022).

Prior studies on the interaction between adults and young children have predominantly concentrated on the “teacher-child” or “parent-child” dyads, with no consideration given to tripartite interactions and inadequate emphasis on parenting education for young children. In parent-child programs, the primary subjects of the activities are teachers, parents, and children, resulting in a multi-directional interaction that is richer than the traditional definition of two-party interaction. The research used a qualitative research methodology to document and analyze the dynamic interactions within parent-child programs, aiming to investigate the characteristics and issues associated with the emergence and evolution of interactive behaviors among teachers, parents, and young children. The early years of a child’s life are foundational for cognitive, emotional, and social development, with the quality of teacher-child interactions playing a critical role in shaping these outcomes. High-quality educational environments, particularly those emphasizing responsive and engaging adult-child interactions, have been found to significantly influence children’s executive functions (Choi et al., 2016; Duval et al., 2016). As demonstrated by Veraksa et al. (2019), the richness of these interactions is closely linked to language acquisition and cognitive growth. Similarly, research by Tonge et al. (2019) emphasizes the importance of physical learning environments such as outdoor settings—in enhancing the quality of interactions and facilitating child engagement.From a developmental trajectory perspective, Abbey et al. (2018) explore how biologic sensitivity and early environmental factors contribute to children’s long-term adaptability and coping strategies. Studies by Liu (1999) and Savova (2022) further underline the importance of pedagogical collaboration among teachers, children, and parents in nurturing holistic child development during the preschool years. Meanwhile, large-scale intervention studies, such as those by Del Boca et al. (2022), provide empirical evidence that structured parenting programs can positively affect family time-use patterns, potentially reinforcing educational gains. Adding a behavioral and interactional lens, Farzana and Blanco (2018) use computational models to characterize social exchanges, aligning with findings by Stefan and Sibilis (2022), who examine nonverbal communication in generative play-based paradigms. Collectively, this growing body of literature supports the premise that intentional, high-quality interactions in early education are essential for optimal child development across multiple domains.

Child development refers to the physical, cognitive, emotional, and social growth that happens from infancy to early childhood. Early childhood education helps to facilitate this development by offering structured learning experiences that encourage curiosity and abilities. Social interaction theories, such as Vygotsky’s sociocultural theory, emphasize the role of connections and communication in molding a child’s learning and behavior. These ideas focus on how interactions with caregivers and peers affect language development, problem solving, and social competency. Together, they provide a framework for understanding how children grow and learn in their social contexts.

The problem of the research topic is that tripartite relationships in Chinese parent–child programs are often rigid and lack real child participation and ineffective communication between teachers and parents. To address this issue, it is essential to investigate the dynamics and challenges of these interactions to develop a more equitable and inclusive framework. The research examined the nature and challenges that arise in tripartite interactions in China. The goal is to enhance the quality of interactions by facilitating meaningful interactions involving instructors, parents, and children - ultimately, to enhance early childhood development.

## 2 Methodology

### 2.1 Participants

Qualitative research does not have a large population from which to draw random samples, nor does it aim to draw generalized conclusions. Interpretivist researchers tend to select each case purposefully and strive for a complete and accurate description of “what happened and how it happened” to answer the research questions (Glesne, 2021). Therefore, the purposive sampling method was used in the research, and four groups of children and their parents from two preschools in Nanjing, China, were recruited to participate in parent–child programs. Following the declaration of the research’s purpose and the acquisition of participants’ permission, a total of 29 children average aged 30 months, along with their parents and four female teachers, participated in the research (see Table 1).

**Table 1:** Research participants.

Institutional attributes	Group	Number of parent and child	Teacher	Years of experience
Preschool 1	Group 1	6 pairs	T1	15 years
	Group 2	9 pairs	T2	10 years
Preschool 2	Group 3	7 pairs	T3	8 years
	Group 4	7 pairs	T4	4 years

Parent–child programs were held once a week for 1 h at a time. Each group conducts 16 parent–child education activities per semester. Both parents and children participate in the program activities under the guidance of the teacher.

### 2.2 Research methods

The research used observation and interview methods to explore the process of tripartite interaction. First, the research used the event sampling observation method, and to better capture the detailed content of the events that occurred, video equipment was used to film the tripartite interaction events during the observation. To lessen the chance of interfering with the interaction process, pre-observation was carried out before formal observation. The filming covered the time, place, language, action, and context of the tripartite interaction event. A total of 1,130 tripartite interaction events were collected from 28 parent–child activities in the research. After acquiring the video recordings of the tripartite contact events, they were immediately categorized into text and numbered according to “time-group-type.”

Secondly, interviews were conducted with parents and teachers based on the current interaction events. Parents participated in both random and semi-structured interviews. Random interviews were conducted in response to the observed interaction events throughout the research, capturing their thoughts and any confusion they experienced during these interactions. Additionally, five parents were intentionally selected for semi-structured interviews to gain deeper insights into their perspectives on the interaction process, their roles, and their children’s development during parent–child activities. Finally, semi-structured interviews were also held with four teachers to gather their interpretations of the interaction events and their perspectives on parent–child activities.

In order to ensure the smooth conduct of the research, consent was obtained from the research participants based on the principle of confidentiality. The basic principles of the research were “respect for the wishes of the researched” and “absolute confidentiality of the privacy of the researched.”

### 2.3 Data collection

The researcher ultimately acquired 1,130 video recordings of tripartite interaction events along with 23 interviews. The transcription and organization of the recorded material involved several steps: initially, the entire video of the interaction process was viewed to identify the participants and content of the events. The video was subsequently re-watched in full, with pauses and replays as needed to pinpoint key initiations, themes, processes, and outcomes of the interactions. Finally, the interactive events were meticulously transcribed. This organization of the video material enables a detailed, iterative, and dynamic analysis of the interactions (Erickson, 1996). During the transcription process, both verbal and non-verbal behaviors were documented sequentially to reduce transcription bias. The interviews were also transcribed, and essential information points were extracted to support the analysis of the recorded data.

### 2.4 Data analysis

To facilitate further analysis and interpretation of the data, transcripts were coded and categorized using NVivo12. Initially, the interaction events were briefly classified. Each interaction initiator had two interaction objects. A tripartite interaction occurs when an initiator selects an interaction object, subsequently involving a third party. Consequently, there are six fundamental types of interactions among the three parties, determined by the initiator and the first interaction. The interaction events are categorized into six types based on the following configurations: “Teacher-Parent–Child (T–P-C), Teacher-Child–Parent (T-C-P), Parent–Child-Teacher (P-C-T), Parent-Teacher-Child (P–T-C), Child-Teacher-Parent (C-T–P), Child–Parent-Teacher (C-P–T).”

Secondly, the interactive events were systematically coded in layers to discern the primary interactive themes, which represented the central issues or content of the events. The coding process commenced with open coding, which involves assigning new concepts to the source material on a sentence-by-sentence basis while setting aside personal biases and maintaining an open perspective. This can be accomplished by employing the original utterance’s native vocabulary or by articulating it with scientifically rigorous terminology (Zhou et al., 2022). Open coding is fundamentally guided by the intent of the interaction initiator or the interaction’s objective, yielding a total of 31 initial concepts through meticulous word-by-word coding until saturation is achieved. Axial coding entails identifying the relationships among the initial concepts derived from open coding, such as causal, structural, typological, processual, and strategic relationships. This phase of axial coding is developed after a thorough examination of each initial concept, leading to the reorganization or amalgamation of various initial concepts. Through a systematic organization and categorization of these initial concepts, the research identified that interactions T-C-P and P-C-T initiated by adults toward children; interactions C-T–P and C-P–T occurring among adults; and interactions T–P-C and P–T-C initiated by children exhibited homogeneity in coding, resulting in the consolidation of the six interaction types into three distinct categories. Finally, based on the axial coding, core categories were selected following a systematic analysis, culminating in six selective codes (see Table 2).

**Table 2:** Thematic coding of tripartite interaction events.

Type of interaction	Open code	Axial code	Selective code
T-C-P&P-C-T	Difficult problems encountered; behavioral guidance and correction	Activity guidance	Education and guidance
	Ask questions; guide children to answer question	Questions and prompts	
	Correct problem behaviors of young children; regulate activities	Regulate behavior	
	Encourage young children to participate in activities	Concern and encouragement	Communication and encouragement
	Attracting teacher/parent attention to young children; evaluating young children’s performance		
	Repeat or illustrate the young child’s ideas	Understanding and interpreting	
	Exchange anecdotes about life; greetings; expressions of comfort	Exchange of emotions	
	Young children seek help when they are in trouble; adults initiate help	Seeking and providing help	Help and Replacement
	Adults replace children in play; stop the behavior of young children’s activities	Replace young children in activities	
T–P-C&P–T-C	Teachers and parents share ways to instruct young children; parents seek guidance from teachers	Help and advice	Concerns and guidance
	Concern or comment on the performance of young children in the curriculum	Concern and comment	
	Communicate about the daily lives of young children; Increase the knowledge of young children’s personality traits	Communicating understanding of young children	Communication and comprehension
C-T–P&C-P–T	Seek adult help; ask questions	Seek guidance	Subjective expression
	Expressing wishes and ideas; attracting adult attention	Expression and performance	

Thirdly, an analysis of the formation and evolution of interactive events will be conducted to assess whether the outcomes of interactive behaviors fulfill the intended purpose of the interaction.

**Figure 1:**
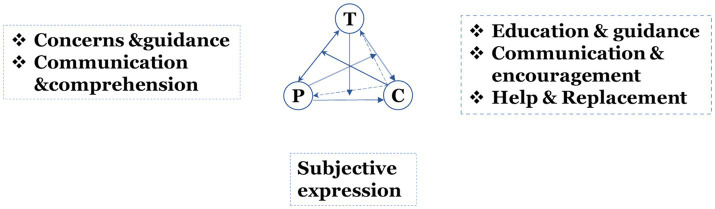
Tripartite interaction model.

After completing the initial analysis, the research constructed a model of tripartite interaction among teachers, parents, and children in parent–child programs. Different lines represent different thematic content, as shown in Figure 1.

Figure 1 depicts six fundamental themes of interactions between children (C), parents (P), and teachers (T): Teaching and correcting children’s behavior is a part of education and guidance. Communication and Encouragement encompasses emotional support, motivation, and appreciation; Help and Replacement shows adults helping children or handing over chores as necessary; Concerns and Guidance refers to conversations between parents and teachers regarding children’s development and teaching strategies; Subjective Expression records children actively expressing their needs, ideas, or inquiries; and Communication and Comprehension entails sharing insights to enhance both parties’ comprehension of the child’s characteristics and behaviors.

## 3 Results

**Figure 2:**
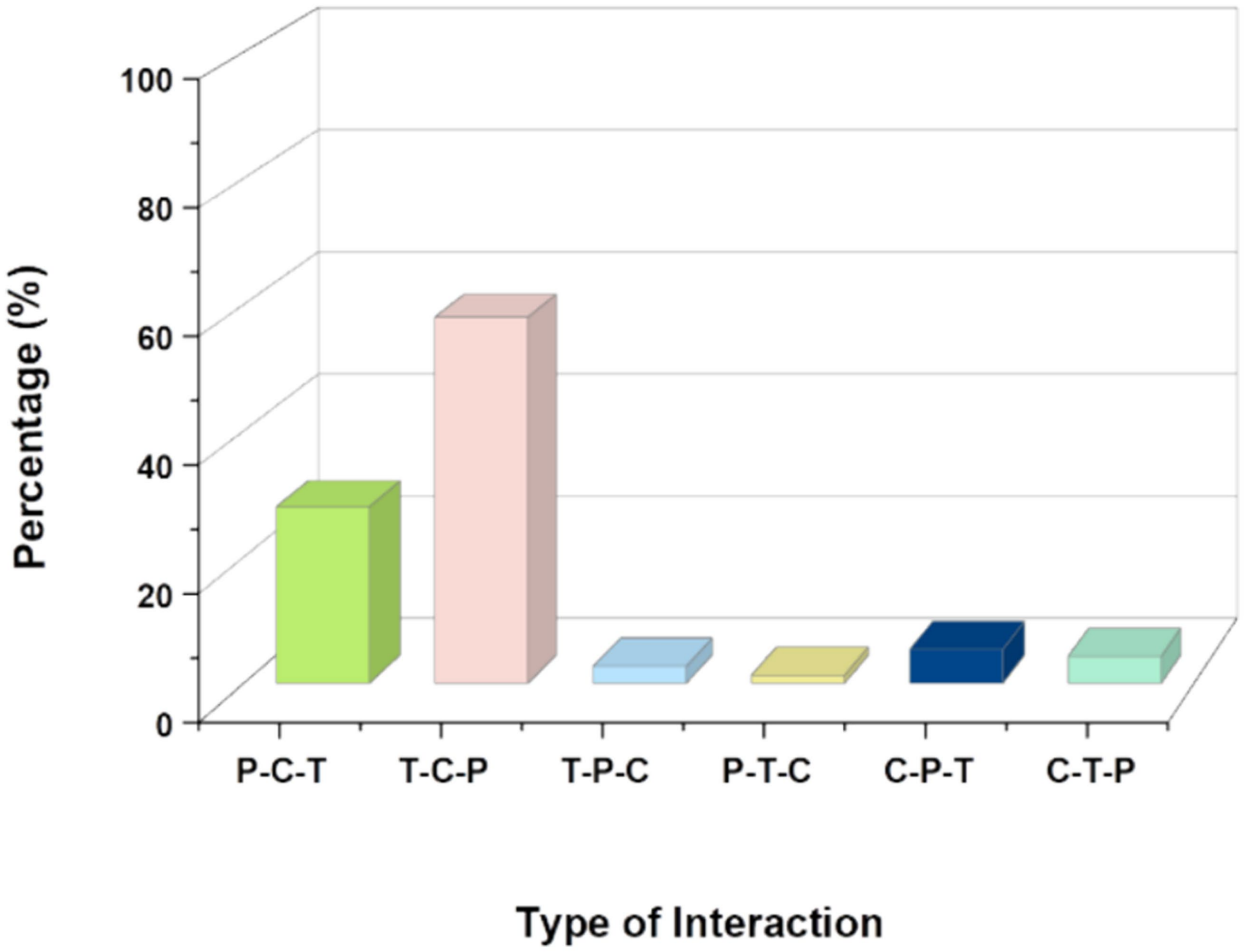
Illustrates the frequency and percentage distribution of tripartite interaction event types in parent–child programs.

As shown in Table 3, the research found that the interaction forms of P-C-T and T-C-P were the main types of tripartite interaction events and together accounted for 86% of the total number of interaction events. Thus the parent–child program was dominated by adult-initiated interactions with young children. Figure 2 illustrates the Frequency and percentage distribution of tripartite interaction event types in parent–child programs.

**Table 3:** Frequency of tripartite interaction event types in parent–child programs.

Type of interaction	Frequency	Percentage (%)
P-C-T	322	28
T-C-P	652	58
T–P-C	31	2.7
P–T-C	15	1.3
C-P–T	61	5.4
C-T–P	49	4.3

### 3.1 Interactive themes

**Figure 3:**
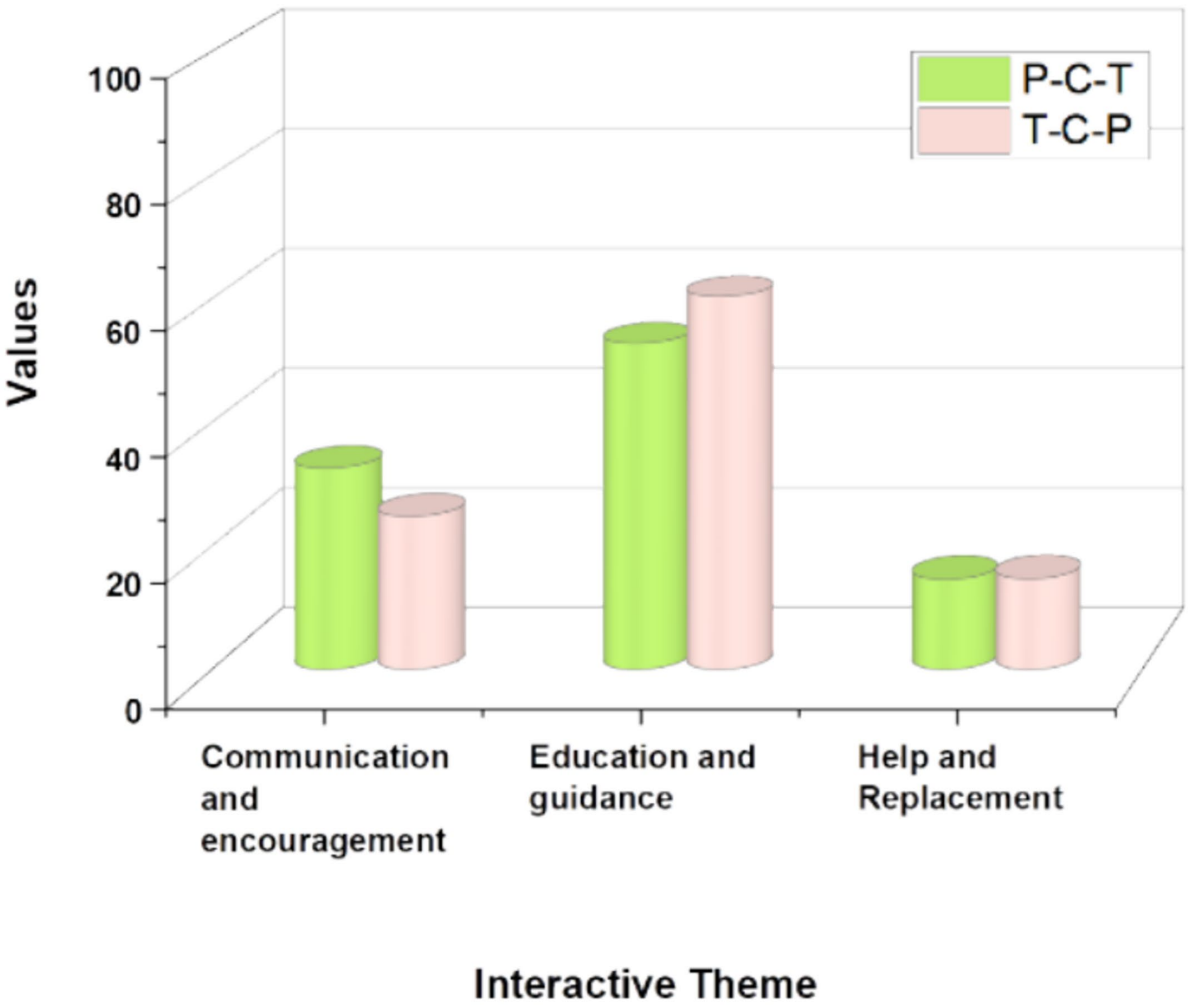
Represents the themes of interactive behaviors in P-C-T and T-C-P tripartite interactions.

The interactive theme determines the direction and content of the participants’ communication, as well as the purpose and form of the event (Nia, D.,2019). As shown in Table 4, the theme of education and instruction was the most frequent among the interactions initiated by adults with young children. The tripartite interactions were focused on achieving the educational purpose of the parent–child program. Figure 3 represents the themes of interactive behaviors in P-C-T and T-C-P tripartite interactions, categorized by communication and encouragement, education and guidance, and help and replacement.

**Table 4:** Themes of P-C-T&T-C-P interactive behavior.

Type of interaction	Interactive theme		
	Communication and encouragement	Education and guidance	Help and replacement
P-C-T	105 (32.6%)	170 (52.8%)	47 (14.6%)
T-C-P	162 (24.8%)	394 (60.4%)	96 (14.7%)

#### 3.1.1 Education and guidance: teacher and parent role-playing

The parent–child program involves achieving the educational objectives of the activity by fostering communication and interaction among the three parties within a specifically designed educational environment. The main way for parents and teachers to achieve the educational purpose of early childhood education is through interaction with the theme of education and guidance. After the teacher explains the arrangement of the activity, the use of materials, or the rules of the game, the parents provide guidance to the children based on their understanding of the purpose of the activity and their children’s current activities. During this process, the teacher observes the parents and their children’s behavior and then provides guidance based on specific problems in a particular parent–child activity that align with the educational objectives. This type of interaction requires mutual understanding and educational consistency between parents and teachers, and both teachers and parents need to have a clear perception of their roles in it.

Symbolic interaction theory suggests that human beings are able to assign symbolic symbols to objective things, human conceptual behaviors, and even physical gestures such as facial expressions and gestures. The symbolic interaction theory ignores how meaning-making processes shape classroom dynamics. It fails to connect these symbolic interpretations to the specific actions seen in the study. A greater amount of complete integration would reveal how students and teachers co-create meaning through interaction (Husin et al., 2021). The consensus of these behavioral gestures in a group makes the group a social community that can communicate and survive with each other (Song, 2007). The symbolic meaning of behavioral gestures allows people to understand and anticipate each other’s behavioral intentions, thus enabling communication between multiple parties (Ibolya, 2010). Mead, G. H., refers to this ability to understand behavioral gestures as role-playing, which is the ability to adapt one’s own behavior in accordance with the attitudes and intentions of others.

In parent–child programs, parents undertake multiple roles as learners, educators, facilitators of teacher-child interactions, and caregivers of young children. Parents act as learners, and the process of learning encompasses various levels. Subsequently, they acquire knowledge of the teaching methods and procedures pertinent to the activity, ultimately grasping the educational objectives and purposes intended to be fulfilled through the play activity. Furthermore, parents serve as educators and possess certain authorities and obligations in many activities. When children face challenges in activities, parents can offer tailored direction through contact, which also impacts the teacher’s educational approach. Third, parents facilitate teacher-child interactions. In contrast to teachers, parents possess a greater familiarity with their children’s experiences. When issues occur between educators and children, parents must assist their children in comprehending the instructor’s requests or aid the teacher in modifying strategies to better suit the child. Ultimately, parents bear the principal responsibility for the toddler’s behavior and the caregiver’s role. Parents are needed to regulate the behavior of young children when problem behaviors occur.

Likewise, educators assume diverse responsibilities in the activities. Teachers function as facilitators of activities, monitors of parent–child interactions, educators of young children, and providers of parent education, excelling in these roles is a measure of their complete instructional competencies. Educators must consistently modify their responsibilities throughout the contact process. The distinct functions of parents and teachers are interconnected, necessitating that both adapt their responsibilities throughout time in response to the demands of interaction and suitable role fulfillment. This is directly pertinent to the attainment of the educational objectives of the parent–child program. The research takes C1 and her father as a case study.

##### 3.1.1.1 Case 1

*Background: Group 4; parent–child painting activity in which parents and children needed to work together to complete a collage. Teacher 4 (T4) observed the child (C1 29 months) and her father cut the paper into strips and started to color the strips with a water-based paintbrush.*

*Dad took the red paintbrush from the table and began painting the paper strips, commenting on the “C1 look” as he went. T4 approached C1 and said to her father, “I’d like to talk to you for a minute.” After the father put down the brush. T4 continued, “When you color the paper, you need to ask C1 what color to choose, and then you can brush it together.” T4 brought over a yellow paint bottle and said, “Come on, C1, hold the brush in your hand, and we’ll paint the color together.” When C1 heard T4’s comments, she went to take her father’s hand, and T4 quickly said, “She wants her dad to stay with her. Daddy, take her little hand.” T4 placed the pen in C1’s hand, and then the father took his hand and held the pen together. T4 patted C1’s head and remarked, “Very good, very good.” Dad grabbed C1’s hand and started coloring. But C1’s arm fully blocked her view. T4 immediately informed the father, “She cannot see.” T4 assisted C1 in tucking her head into the father’s arm.*

During the aforementioned interactive event centered on “education and guidance,” the father encountered difficulties in assisting his child with drawing, prompting the teacher to participate and offer help to them. The objective of the parent–child painting activity is to enable children to recognize colors and develop their fine motor skills. T4 tried to correct the father’s substitution of C1’s coloring, but she did not explain the value of the activity to the child during the instruction and was still substituting and manipulating the child with her own ideas. When discussing this interactive event, T4 said, “*Children can color paper independently. When I observed the parent coloring instead of the child, I guessed that the child might be experiencing difficulties and that is why I asked the dad to take her coloring with him*.” T4 identified communication challenges with grandparents and fathers due to age and gender disparities, respectively. This indicates that to achieve the interactive objectives of the theme “Education and Instruction,” educators must possess robust communication and guidance abilities and effectively engage parents as collaborators. Educators and parents can form a coalition by effectively assuming various roles in their interactions, thereby facilitating seamless communication and exchange among the three parties.

##### 3.1.1.2 Case 2

*Background: Group 3; 7 children and their parents were seated around a large table with T3 standing at one end of the table, demonstrating how to separate water and oil.*

*T3 first drew a sun shape on white paper with a white oil stick, then showed it to the group and asked, “Children, can you see it?” The child (C5), sitting closest to the teacher, looked at the paper and answered, “It’s a Sun.” Instead of responding to C5, T3 asked the group again, “Can you see it?” C5’s mother patted her and said, “The teacher is performing a magic trick; can you see it on the white paper? There should be nothing on it, right?” T3 picked it up again, showed it to everyone, and said, “C5 can see the sun, but I do not think I can see it. I merely utilized a white paint stick to draw on white paper, rendering it should be invisible to you.”*

This interactive case presents a scenario similar to the “Emperor’s New Clothes.” The adult denies the child’s fact-based response and defers to the teacher’s “authority.” In Mead’s theory of interaction, human beings are able to read each other, anticipate each other’s responses, and adapt to each other in their interactions. Interpersonal communication and interaction occurs through reading and interpreting the gestures of others. Mead calls this ability “feeling and understanding others’ roles” or role apprehension, i.e., the ability to act in response to others’ attitudes and intentions (Song, 2007). T3 designed a “magic” session in which the children’s responses deviated from the teacher’s design, but the teacher chose to continue with her own prediction. C5 understood the teacher’s intention, so she acted as a facilitator in the teacher-child interaction, helping the teacher to correct the children’s responses. However, during this interaction, C5’s ideas were denied and subjectivity was ignored.

#### 3.1.2 Concerns and guidance: teachers and parents develop together through interaction

**Figure 4:**
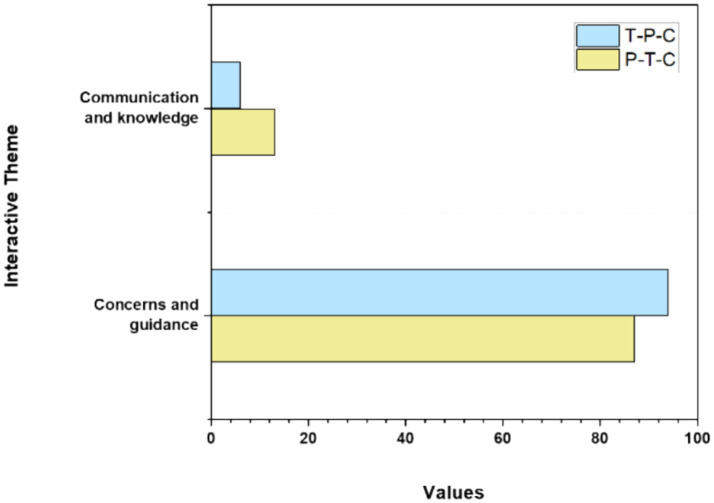
Themes of interactive behaviors in P–T-C and T–P-C tripartite interactions.

Table 5 illustrates that the primary theme of P–T-C and T–P-C interactions pertains to concerns and guidance. Teachers serve as designers and organizers of parent–child activities, leading parents to occasionally seek their assistance regarding children’s issues or their perspectives on children’s performance. Conversely, when the teacher identifies challenges faced by parents and children during activities, the teacher will proactively offer timely assistance. Figure 4 represents the themes of interactive behaviors in P–T-C and T–P-C tripartite interactions, showing proportions of concerns and guidance versus communication and knowledge.

**Table 5:** Themes of P–T-C&T–P-C interactive behavior.

Type of interaction	Interactive theme	
	Concerns and guidance	Communication and knowledge
P–T-C	13 (87%)	2 (13%)
T–P-C	29 (94%)	2 (6%)

##### 3.1.2.1 Case 3

*Background: During a game session at a public parent–child institution, T1 arranged two games, including kicking a ball to shoot at a goal and sending a small animal home along a route. Parents and teachers watched and guided children as they played the games voluntarily.*

*The child (C2) kicked the ball into the archway and then went underneath the archway to pick up the ball, almost knocking over the archway. C2’s grandmother, sitting on the sidelines watching, pointed at C2 and said to the teacher, “This child went underneath.” C2 stayed in the archway to pick up the ball, and the children behind him formed a long queue to wait for him. T1 squatted down and picked up C2’s ball from the other side of the archway, and C2 saw it and immediately climbed out of it. The teacher said to him, “Go and play with the animals and send them home.” The grandmother heard this and said to C2, “Yes, send the little animal home.” But C2 turned around and went back to the arch. When the teacher saw this, she pointed to the little animal and told C2 again, “The bunny is waiting for you to take it home.” C2’s grandmother handed him a basket with a bunny in it, and C2 took the basket and turned around to play another game.*

Confronted with her child’s defiance of game rules, the grandmother is uncertain on how to address the situation and seeks advice from the teacher. T1 indirectly motivates the parent to engage in an alternative game by proposing an appropriate option for C2. During the interview, T1 indicated that while the parent–child program established educational objectives for parents in lesson preparation, teachers encountered challenges in employing suitable communication strategies with various parents during implementation.

#### 3.1.3 Expression of young children’s thoughts

**Figure 5:**
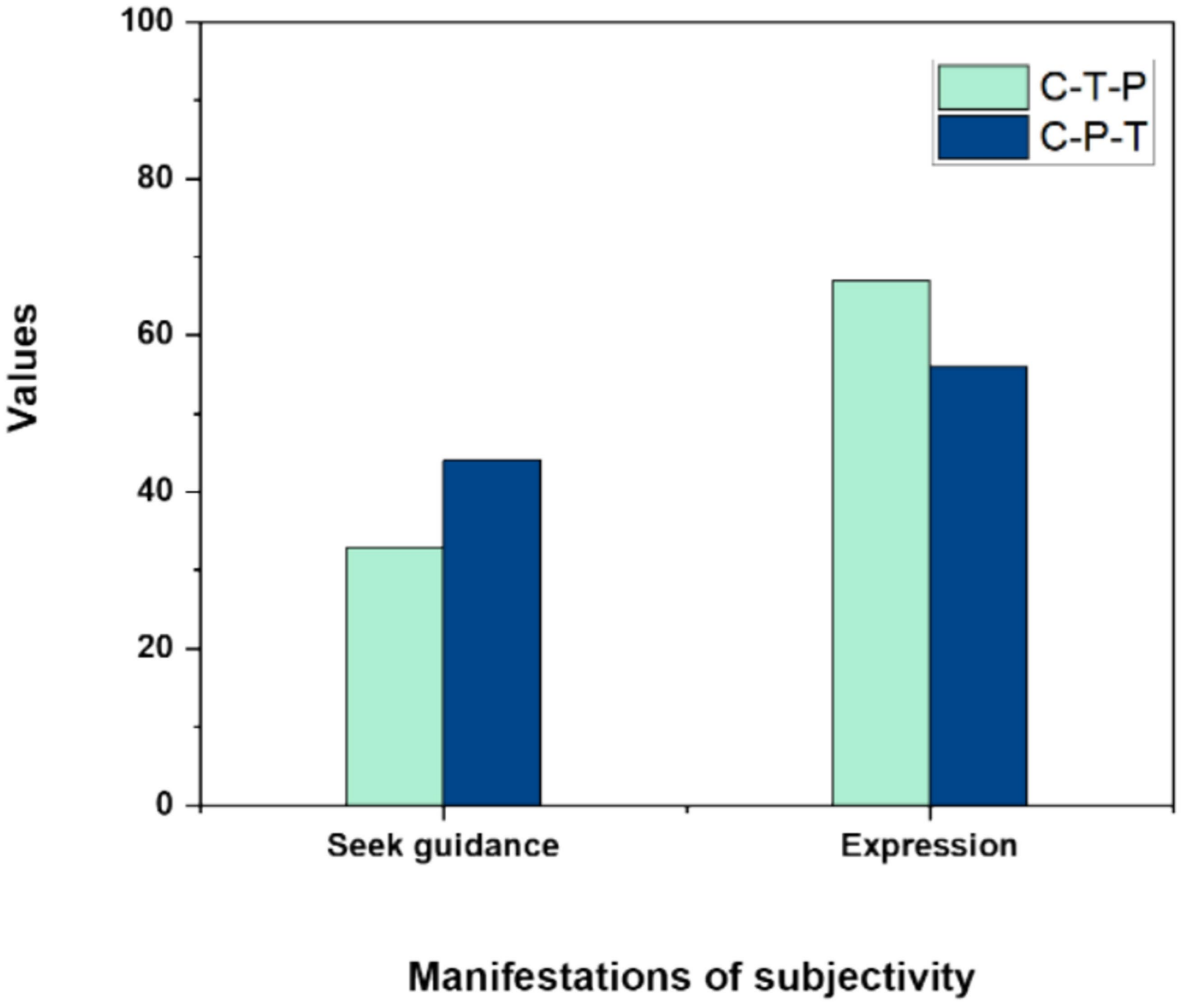
Manifestations of subjectivity in C-T–P and C-P–T tripartite interactions.

The majority of interactions initiated by young children serve to articulate their thoughts and to solicit attention and assistance from adults. This technique exemplifies the children’s subjectivity, showcasing their distinct personalities and enabling parents and teachers to gain deeper insights into the youngsters. Table 6 indicates that children engaged in more tripartite interactions with parents than with teachers, with both interactions predominantly centered on the theme of “Expression.” Figure 5 illustrates the manifestations of subjectivity in C-T–P and C-P–T tripartite interactions, differentiated by seeking guidance and expression.

**Table 6:** Themes of P–T-C&T–P-C interactive behavior.

Type of interaction	Manifestations of subjectivity	
	Seek guidance	Expression
C-T–P	16 (33%)	33 (67%)
C-P–T	27 (44%)	34 (56%)

##### 3.1.3.1 Case 4

*Background: Just after Children’s Day, before the activity began, T2 shared information with the parents and children in the class about the gifts the children had received. Some parents mentioned that their children had received building blocks. T2 informed the parents that “building blocks allow children to use their hands and minds.”*

*When the child (C5) heard what the teacher and parents said, she immediately said out loud to the teacher, “Blocks mean you can build lots of houses.” After that, C5’s voice quickly decreased, and she was still saying something, but the teacher and parents looked at her and did not say anything. T2 smiled, nodded to C5, and then addressed the group, saying, “Let us start today’s activity.” C5 was still muttering to herself, and the grandmother put her finger to her mouth and said, “Shhhh!*”

Adults’ talk about blocks triggered memories of young children’s experiences of playing with blocks. The child was able to initiate the expression of her ideas, but the teacher and parents did not respond appropriately to the child’s ideas or support the encouragement of the child’s expression. The teacher’s primary goal was to continue the teaching activity, while the parents, sensing the teacher’s intention, collaborated with the teacher to regulate the child’s behavior and halt the child’s expression. The teacher’s focus on the predetermined educational task outweighed her concern for the children’s experience, and the purpose of the interaction initiated by the children was not achieved.

### 3.2 Interaction results

Bourdieu considers the field a space of objective relations, with its own logic and necessity. The Bourdieu’s concept of the field does not delve into how power dynamics and social structures impact educational practices. It fails to link the “field” logic to the repetition of inequities observed in classroom interactions. Deeper engagement would improve the analysis of systemic influences on learning settings (Bourdieu and Wacquant, 1992). This space is a synthesis of the network of social relations formed by different individuals and the various social forces and factors they represent (Wang, 2012). The educational environment of parent–child activities is a small social field where three individuals with different identities interact in a complex network of relationships. Each of the three parties possesses different strengths and capitals, which, to some extent, also influences their role positioning. Teachers in the parent–child activity field have more cultural capital than parents and children and thus have relative authority and dominance in the field. And young children are the weakest of all. The “power” of the young child depends on the respect that parents and teachers have for the young child’s subjectivity. The field is also a space of contestation, where the perspective taken by an individual depends on the position he or she occupies in the field, and the contestation and struggle for this perspective arise from the asymmetry of resources and power between the various positions (Bourdieu and Wacquant, 1992). The outcome of the tripartite interaction in the parent–child classroom is produced by the coordinated resistance between the three parties. When the initiator’s intention is accepted and suggestions are adopted, the interaction’s purpose is achieved and vice versa. According to the theme of interaction behavior and whether its purpose is reached or not, they are named as acceptance and rejection, respectively. Table 7 illustrates interactions initiated by young children exhibited the lowest acceptance rate, while C-T–P had a slightly higher rejection rate than C-P–T.

**Table 7:** Overall results of tripartite interaction in parent–child activities.

Result	P-C-T		T-C-P		P–T-C		T–P-C		C-T–P		C-P–T	
	F	P (%)	F	P (%)	F	P (%)	F	P (%)	F	P (%)	F	P (%)
Accept	291	90	565	87	14	93	29	93	30	61	50	82
Reject	31	10	87	13	1	7	2	7	19	39	11	18

The subsequent two cases exemplify the acceptance and rejection of the interaction’s objective, respectively.

#### 3.2.1 Case 5

*Background: In group 3, during a pizza-making activity using paper clay, parents and child needed to press the paper clay into flat “pies.”*

*The child (C6) pressed very hard and carefully, and C6’s mother asked him, “Do you want me to help you?” When the teacher (T3) heard this, she also said to C6, “It’s okay if you let your mom help you press it.” When C6’s mom stretched out her hand and was about to press, she asked again, “Do you want me to help you?” C6 said, “No.” C6’s mom replied “Okay, then press it yourself.” C6 patted the paper clay hard and his mom encouraged him by saying “Harder and harder.” After the child taped a few times, T3 said to C6 “You need Mommy’s help to make your pizza a little bigger, it’s too small to eat.” C6 stopped tapping and looked down in thought, C6’s mom reached up again, “I’ll help you make it bigger, look at me.” C6 watched silently without saying a word.*

In this case, during a parent-initiated tripartite interaction on the theme of “help and replacement,” T3 repeatedly persuaded parents and children to compromise, based on her strong opinions. T3’s main criterion for guidance was the objective of the activity, which was to flatten the paper clay as quickly as possible, and she responded in a randomized interview after the activity, “*If we do not permit parental assistance, we will lack sufficient time for the remainder of the activity.*” While parents were initially able to consciously respect their child’s wishes during the interaction, they were later they were compelled to compromise with the teacher’s recommendations.

#### 3.2.2 Case 6

*Background: In group 4, parents and children needed to decorate elephants with latex adhesive fabric. T4 observes the child (C7) squeezing latex from a latex bottle while his grandmother looks at him.*

*C7 holds the bottle in both hands and squeezes it on the baseboard while his grandmother stares at him. T4 encourages C7 by saying, “C7 is squeezing the bottle very well; keep up the good work.” C7 squeezed out a long strip of latex. Grandma said to him, “You’re squeezing too much.” T4 heard him and said to them, “It’s okay; it’s just a matter of doing it yourself. You can go ahead and put your favorite fabric on it.” C7 chose a piece of cloth and put it on. C7 picked up the bottle of latex and squeezed a little bit to the left side of the elephant, then grandma grabbed his latex and said, “You do not want to squeeze any more, you are squeezing too much.” C7 turned to face Grandma and remarked, “That’s okay.” Grandma spread out the latex he had just squeezed and asked, “Why do you say it’s okay?” T4 turns away.*

Parental support for teacher-initiated interactions designed to promote children’s independence was lacking, as the grandmother perceived issues with her kid’s movement. The grandma aimed to conserve supplies, whereas the teacher sought to enable the child to independently complete the task and gradually develop hand strength during manipulation. Nonetheless, T4 failed to adequately communicate the intent of his interaction to the grandmother, resulting in a conflict between the parents’ perspective and the needs of the kid and teacher, thus preventing the fulfillment of the interaction’s objective. The objective of the tripartite interaction is contingent upon the contest for “power” among the three participants.

## 4 Discussion

### 4.1 Monolithic interaction structure and insufficiently considers children’s subjectivity

In classroom research, greater emphasis has been placed on the “participation framework” of teacher-student interaction. Kazden asserts that the predominant discourse structure in conventional classroom instruction is the IRE sequence, which comprises teacher initiation, student response, and teacher evaluation. The application of Kazden’s IRE paradigm is superficial, lacking an in-depth exploration of why this discourse pattern persists and how it affects student engagement. The critique would benefit from examining the constraints teachers face when employing the IRE sequence, as this would offer valuable insight into the structural factors shaping interaction patterns (Tian and Huang, 2019). The IRE model of verbal interaction employed to investigate teacher-student dynamics is predominantly teacher-driven, regulating both the topic and the frequency of engagement, while disregarding student discourse. This dominant structure of interaction is also reflected in the research’s parent–child program, in which the teacher (parent) initiates, the child reacts, and the teacher (parent) evaluates. The main purpose of I-R-E interaction is to input educational content to the child and to achieve the educational goals set out in the curriculum. This type of interaction faces the following challenges:

Primarily, teachers have overlooked the dual-subject educating objective of the parent–child program. The significance of parent–child programs lies not only in fostering the healthy physical and mental development of children and toddlers but, more crucially, in augmenting parents’ comprehension of the developmental traits of young children and enhancing their capacity to offer educational guidance. In the I-R-E discourse paradigm, teachers neglected direct advice and influence on parents, resulting in insufficient interaction between parents and teachers.

Secondly, children have forfeited their subjectivity. Subjectivity is the conscious, independent, dynamic, and creative personality of an individual that evolves via interaction with an object. Subjectivity in developmental psychology refers to children’s increasing internal capacity to perceive, understand, and interact with their environment as autonomous individuals. Based on Vygotsky’s sociocultural theory, children’s subjectivity is developed through social contact and mediated by language, tools, and symbols. Piaget’s constructivist approach emphasizes that children actively generate knowledge by exploring and manipulating their surroundings, strengthening the relationship between subjectivity and autonomy (Wang, 2019). Autonomy and subjective initiative underpin creativity, which is defined by exploration and innovation, representing the pinnacle of individual subjectivity. Children initiate interactions using verbal attempts, gestures, and expressions to communicate their needs and interests. However, adults frequently restrict children’s expressions through quick appraisal, topic diversion, or non-acknowledgement, limiting their agency. When adults respond with encouragement, open-ended questioning, and validation, children’s self-expression grows rapidly, resulting in more balanced and dynamic three-way conversations. Children frequently employ subtle signs such as eye contact, reaching out, or vocalizing to attract and engage others. They perform experiment with language, tone, and nonverbal signs to see how adults respond to their demands. However, their expressions are frequently limited by adults’ controlling activities, such as interrupting, redirecting the conversation, or choosing adult agendas, which may reduce or hide the child’s voice. In constrast, Supportive adults allow children to take the initiative by patiently listening, following the child’s lead, and recognizing their efforts, resulting in a more authentic and reciprocal interaction dynamic. Developmental psychology recognizes young children’s creativity as an expression of their symbolic thinking, problem-solving skills, and imaginative abilities—processes that are fundamental to both cognitive and emotional development (Bruner et al., 2000). When youngsters are encouraged to freely express themselves, they are more likely to participate in divergent thinking, which is an essential component of creativity. Children’s subjectivity refers to their ability to actively participate and express themselves as self-sufficient persons. Recognizing this subjectivity is essential for meaningful relationships that respect people’s views and feelings. When acknowledged, it promotes children’s growth and participation. Ignoring it may limit their agency and impede successful communication in educational contexts. Furthermore, children’s subjectivity highlights their role not as passive beneficiaries of adult supervision, but as active participants in their learning contexts. It involves recognizing their emotions, ideas, and viewpoints as legitimate and influential in creating educational experiences. Fostering children’s subjectivity promotes autonomy, critical thinking, and self-confidence, which are essential for their overall development. This understanding assists early parenting programs in bridging the communication gap between adults and children, resulting in more responsive and adaptive interactions. Finally, recognizing children’s perspectives creates the groundwork for respectful and empowered educational practices (Yang, 2015). Children’s creativity primarily manifests in creative thinking and practical skills. In the I-R-E discourse paradigm, educators and parents consistently influence children’s methods toward predetermined objectives, thereby failing to genuinely honor children’s subjectivity.

Furthermore, the interactions initiated by children resulted in the highest rate of rejection. During these interactive events, children’s thoughts were suppressed, resulting in their turn into passive learners. Consequently, addressing the interplay among multiple subjects and establishing the subject position of young children is an issue that requires further investigation.

### 4.2 Communication between teachers and parents is inadequate and superficial

In parent–child programs, parents serve as secondary educators, necessitating tight collaboration between parents and instructors. However, communication in existing parent–child programs is evidently insufficient. Teachers’ instruction to parents pertains to the interpersonal dynamics among adults. Throughout the interview with T3, it was stated that “when a parent was focused on his or her cell phone during the activity, my strategy was to engage with his or her child and make requests to the child to redirect the parent’s attention toward the child. I believe that stating, ‘So-and-so parent, you must listen to this with your child,’ will result in him losing face.” Face is a prevalent psychosocial phenomenon in Chinese interpersonal relationships, and its cultural rationale endures even in educational contexts. The concept of face signifies a favorable perception of one’s social identity, and in an educational setting with numerous parents present, a teacher who publicly admonishes or corrects a parent leads to that parent experiencing a loss of face. This exemplifies the nature of Chinese interpersonal relationships, resulting in much confusion between educators and parents.

Cultural psychology holds that psychological processes are firmly embedded in societal environments. In China, the concept of “face” (mianzi), interpersonal harmony, and hierarchical relationships are based on Confucian values that emphasize respect for authority, collectivism, and the preservation of social harmony. These cultural standards influence both vocal and nonverbal communication in educational contexts. The research obtained a better understanding of the underlying motivations that drive behavior in educational settings by incorporating cultural psychology. For example, teachers’ reluctance to publicly correct parents is often motivated by a desire to maintain social harmony and avoid losing face. This dynamic generates a communicative environment in which indirect techniques are preferred over confrontation. Furthermore, such cultural conventions could prevent open exchanges between parents and teachers, restricting the extent of their collaboration. Understanding these culturally grounded behaviors allows for the creation of more context-sensitive instructional practices. As a result, cultural psychology offers a useful interpretive framework for assessing and developing interpersonal dynamics in Chinese parent–child programs.

Furthermore, teacher-parent connection is fundamentally a social engagement facilitated by the child. The presence and preservation of this interaction rely on the attitudes, emotions, trust, and commitment of both parties involved (Zhang, 2020). Interviews with parents revealed a deficiency in the emotional connection between certain parents and teachers. Parents, when faced with challenges, would attempt to resolve them independently or seek assistance from fellow parents, preferring not to bother the teachers.

Positive examples of cooperation included parents actively supporting teachers’ supervision while encouraging children’s autonomy, resulting in a collaborative environment. Children’s natural contributions were occasionally encouraged, promoting mutual engagement and shared learning among all participants. For example, parents were frequently observed mimicking instructors’ educational practices at home, reinforcing consistency in children’s behavior and learning. This alignment between home and school resulted in a pleasant learning environment that emphasized mutual respect and academic values.

Furthermore, teachers frequently acknowledged and commended children’s efforts, which prompted parents to repeat the positive reinforcement, resulting in a supportive triangle of encouragement. In various activities, children were observed initiating interactions that brought teachers and parents together, such as asking questions or seeking assistance with tasks. These instances highlighted the children’s rising confidence and the safe environment generated by positive collaboration. Teachers and parents also worked together to develop solutions to behavioral concerns, demonstrating collaborative problem-solving rather than unilateral correction.

The informal emotional exchanges in the triad were widespread, such as laughing together, expressing pride, or comforting one another, which helped to strengthen relationships. Teachers frequently referenced parental insights to personalize their instruction, and parents responded by implementing teacher-recommended practices at home. The cooperative dynamic was most visible in complex activities, when parents supplied scaffolding just when necessary, allowing children to explore independently under modest monitoring. These examples demonstrate that the research does not simply focus on inadequacies, but also acknowledges the positive synergy and co-construction of meaning in tripartite relationships, which are critical for holistic development.

In addition, the guidance provided by teachers to parents was superficial. In parent–child programs, educators provide parents with a succinct explanation of the program’s educational importance for children’s development. However, the instructors’ explanations often use technical terms like “vestibular sensation” and “sensory training,” which require a basic understanding of psychology for comprehension. During the interview with the parent (P5), she stated, “At times, I do not comprehend what the teacher is conveying, yet I must prioritize my child’s needs.” Consequently, the interaction between educators and parents remains formal and superficial, leaving parents largely uninformed about their children.

Furthermore, the research identified effective instances of beneficial three-way interactions that enhanced the learning experience. The theme of the influence of Chinese cultural background on communication style is important because it impacts how parents, teachers, and children interact with one another. Indirect communication, respect for hierarchy, and conflict avoidance are all strongly established in Chinese social relationships. These cultural standards frequently result in complex, nuanced interactions that differ from Western communication patterns. Understanding these cultural foundations can provide further insight into the dynamics of tripartite interactions, as well as support in the development of culturally sensitive and effective educational programs. Additionally, the influence of the Chinese cultural background on communication techniques such as the concepts of “face” and harmony is significant and deserves further investigation in future research.

### 4.3 Tripartite interaction for mutual growth

Previous studies on interaction have primarily concentrated on the development of knowledge experiences in children, with less analysis on the collaborative enhancement of knowledge experiences among numerous participants in the interaction. The examination of tripartite interaction demonstrates the existence of socially constructed learning experiences inside the relationship.

The teacher engages in the parent–child interaction. This interactive learning approach, namely P-C-T and C-P–T, offers a more individualized method of experiential construction. In this educational style, the teacher assumes the role of an observer and supporter; by assessing the needs and challenges in parent–child interactions, the teacher can offer focused guidance to facilitate the growth of both parents and children. Meanwhile, parents’ essential educational methodologies and notions motivate teachers and enhance the breadth and depth of their experience.

Secondly, parents participate in the teacher-child relationship, specifically T-C-P and C-T–P. In this educational approach, parents function as observers of their children’s learning, supporters of educators’ instruction, and learners who comprehend the teaching attempts of the teachers. The various roles serve as a challenge for parents. Parents must establish connections between children and instructors when interactions are challenging. Parents will assist teachers in comprehending children’s perspectives while simultaneously translating the teacher’s instruction into a more accessible language for children. Parents serve as the child’s emotional and psychological sanctuary, fostering a secure and conducive psychological atmosphere for interactions between the child and the instructor, so enhancing the child’s drive to engage in classroom activities. Nonetheless, it was observed that several parents were not actively engaged in the teacher-child relationship and required prompting from the teacher to participate. Passive parents engaged in teacher-child interactions had a diminished sense of role; nonetheless, they derived benefits from their involvement in the interaction process.

Finally, children participate in parent-teacher interactions, namely P–T-C and T–P-C. During this process, parents and educators exchange insights regarding the child’s observations, thereby enhancing educators’ comprehension of the child’s personality, interests, and needs. Parental confusion is also communicated, and educators provide parents with specific guidance. These diverse tripartite interactions address the needs of children while simultaneously prompting instructors and parents to recognize deficiencies in the planning and operation of programs, thus contributing to the development of all three parties.

## 5 Conclusion

The aforementioned research and analysis indicate that tripartite interaction in parent–child programs significantly supports children’s development and enhances the educational literacy of parents and teachers; however, certain aspects of this interaction require improvement and enhancement. Consequently, educators ought to consider the subsequent advice while executing parent–child programs: The design of parent–child programs should prioritize dual objectives. Parental educational objectives should be formulated in alignment with children’s activities. Parents must clearly understand their roles in parent–child programs, and therefore, they should be educated in essential parenting knowledge and pedagogical abilities. Secondly, parent–child programs must align with the children’s age and interests. The curriculum must leverage children’s life experiences and provide sufficient flexibility to facilitate the prompt modification of content in accordance with activities. In the structured framework of interactive classroom engagement, educators and parents collaborate to uphold a designated environment, which is preordained by the teacher according to a systematic instructional design (Erickson, 1996). When certain parents and children respond throughout the activity in a manner that deviates from the teacher’s established script, the original script must be abandoned, and a new dialogue should be initiated. Gutierrez et al. (1995) introduced the notion of “third space” as a social environment where individuals can abandon ingrained scripts (such as the teacher’s predetermined educational objectives in the case study) and restructure the activity according to the children’s experiences. Educators must be cognizant that interaction transcends mere discussion among individuals; it constitutes an intrinsic dialogue among dynamic participants, wherein children and parents are regarded as agents of change rather than passive listeners. The benefits of numerous contacts should be fully leveraged to provide a robust educational environment for parent–child development.

Finally, teachers, parents, and children are all subjects in tripartite interaction; therefore, they must acknowledge and respect one another’s subjectivity. Specifically, parents provide children with greater autonomy during activities and promote the articulation of their thoughts. This, in turn, promotes the establishment of equitable, harmonious, and advantageous relationships between the subjects.

### 5.1 Limitations and future direction of the research

The research selected four parent–child courses from two institutions as case study subjects, which limits generalizability; thus, future large-sample investigations on the state of full tripartite contact are necessary. The primary objective of the research on tripartite interaction in parent–child activities is to identify strategies that foster the development of all three parties. Consequently, future research could employ action research methodologies and collaborate with parent–child education institutions to enhance practical activities for parents and children. The research pointed out that children’s subjectivity is not only neglected, but intentionally suppressed in adult-driven I-R-E interactions. This relationship turns children become passive responses, reducing their agency and inhibiting innovation. Future research could examined into inclusive practices that empower children as equal participants in educational discourse.

## Data Availability

The original contributions presented in the study are included in the article/Supplementary material, further inquiries can be directed to the corresponding author.
