# Supplementary material for: Tripartite interaction among teachers, parents and young children in Chinese parent–child program
Source: Front Psychol. 2025 Aug 19;16:1602763. doi: 10.3389/fpsyg.2025.1602763 (PMC12402806; doi:10.3389/fpsyg.2025.1602763)
Supplement: Supplementary file 1 [file Supplementary_file_1.docx]

T-C-P

## Codes

| **Name** | **Description** | **Sources** | **References** |
| --- | --- | --- | --- |
| Education and guidance | Teachers observe parents and children encountering questions or difficulties during manipulative activities or games, and then provide guidance to the parent-child pair during the manipulative activities or supplement and correct the parent's guidance of the child. In this process, parents and teachers understand each other and work together to provide support and assistance in guiding the child. | 28 | 394 |
| Activity Guidance |  | 28 | 71 |
| Difficult problems encountered |  | 16 | 30 |
| behavioral guidance and correction |  | 22 | 41 |
| Questions and prompts | Parents and teachers, as educators, purposefully convey educational content to young children, consolidate what they have learned, test their learning outcomes, and therefore ask them questions. | 28 | 266 |
| Ask questions |  | 28 | 145 |
| guide children to answer question |  | 28 | 121 |
| Regulate behavior |  | 23 | 57 |
| Correct problem behaviors of young children |  | 20 | 38 |
| Regulate activities |  | 22 | 19 |
| Communication and encouragement | Encouraging parent-child participation is when parents or young children are not interested in an activity, the teacher leads the parent and child to work together to find ways to encourage the child to participate in the activity. | 28 | 162 |
| Concern and encouragement |  | 28 | 103 |
| Encourage young children to participate in activities |  | 26 | 51 |
| Attracting teacher/parent attention to young children |  | 24 | 26 |
| Evaluating young children's performance |  | 20 | 26 |
| Understanding and interpreting |  | 27 | 43 |
| Repeat or illustrate the young child's ideas |  | 27 | 43 |
| Exchange of emotions | Parents, children and teachers simply communicate interesting things that have happened in their lives or express their emotions verbally or nonverbally, without any thoughts of activity guidance or commentary. This kind of interaction mostly happens before or after class, when parents and children greet their teachers and exchange stories about interesting things that have happened in their lives. It also includes the attention paid by parents and teachers to humorous incidents involving young children in the classroom, as well as young children's desire to receive emotional reassurance and affirmation from their parents. | 11 | 16 |
| Exchange anecdotes about life |  | 4 | 4 |
| greetings |  | 10 | 10 |
| expressions of comfort |  | 2 | 2 |
| Help and Replacement | During parent-child class activities, when young children encounter difficulties with their work, or when adults think that the children are not doing a good job, have problems with incorrect work, are slow, etc., parents cannot help but help the children instead. Teachers either stop them or tacitly support the parents' substitution. Apart from parents, teachers sometimes become substitutes as well. | 26 | 96 |
| Seeking and providing help |  | 28 | 60 |
| Young children seek help when they are in trouble |  | 16 | 22 |
| adults initiate help |  | 20 | 38 |
| replace young children in activities |  | 17 | 36 |
| Adults replace children in play |  | 15 | 19 |
| stop the behavior of young children's activities |  | 16 | 17 |
|  |  |  |  |

1. C-T

| **Name** | **Description** | **Sources** | **References** |
| --- | --- | --- | --- |
| Education and guidance |  | 28 | 170 |
| Activity Guidance |  | 28 | 145 |
| Difficult problems encountered |  | 24 | 87 |
| behavioral guidance and correction |  | 21 | 58 |
| Questions and prompts |  | 6 | 7 |
| Ask questions |  | 4 | 2 |
| guide children to answer question |  | 5 | 5 |
| Regulate behavior |  | 14 | 18 |
| Correct problem behaviors of young children |  | 10 | 8 |
| Regulate activities |  | 11 | 10 |
| Communication and encouragement |  | 28 | 105 |
| Concern and encouragement |  | 28 | 89 |
| Encourage young children to participate in activities |  | 25 | 57 |
| Attracting teacher/parent attention to young children |  | 13 | 15 |
| Evaluating young children's performance |  | 15 | 17 |
| Understanding and interpreting |  | 6 | 7 |
| Repeat or illustrate the young child's ideas |  | 6 | 7 |
| Exchange of emotions |  | 9 | 9 |
| Exchange anecdotes about life |  | 3 | 3 |
| greetings |  | 4 | 4 |
| expressions of comfort |  | 2 | 2 |
| Help and Replacement |  | 22 | 47 |
| Seeking and providing help |  | 19 | 16 |
| Young children seek help when they are in trouble |  | 14 | 7 |
| adults initiate help |  | 13 | 9 |
| replace young children in activities |  | 21 | 31 |
| Adults replace children in play |  | 17 | 20 |
| stop the behavior of young children's activities |  | 13 | 11 |

T-P-C

| **Name** | **Description** | **Sources** | **References** |
| --- | --- | --- | --- |
| Concerns and guidance |  | 13 | 29 |
| Help and advice | Parents and children encounter difficulties and problems during activities, and the teacher provides help and advice to the parents. Instead of providing direct guidance to young children, they are more passively involved in parent-child communication. | 11 | 19 |
| Teachers and parents share ways to instruct young children |  | 9 | 10 |
| parents seek guidance from teachers |  | 8 | 9 |
| concern and comment | The teacher pays attention to and comments on the performance of young children in their activities, and communicates with parents about their children's learning lives. | 9 | 10 |
| Concern or comment on the performance of young children in the curriculum |  | 9 | 10 |
| Communication and comprehension |  | 2 | 2 |
| Communicate about the daily lives of young children |  | 1 | 1 |
| Increase the knowledge of young children's personality traits |  | 1 | 1 |
|  |  |  |  |

P-T-C

| **Name** | **Description** | **Sources** | **References** |
| --- | --- | --- | --- |
| Concerns and guidance |  | 13 | 13 |
| Help and advice |  | 11 | 11 |
| Teachers and parents share ways to instruct young children |  | 5 | 8 |
| parents seek guidance from teachers |  | 3 | 3 |
| concern and comment |  | 2 | 2 |
| Concern or comment on the performance of young children in the curriculum |  | 2 | 2 |
| Communication and comprehension |  | 2 | 2 |
| Communicate about the daily lives of young children |  | 1 | 1 |
| Increase the knowledge of young children's personality traits |  | 1 | 1 |
|  |  |  |  |

C-T-P

| **Name** | **Description** | **Sources** | **References** |
| --- | --- | --- | --- |
| Subjective expression |  | 21 | 49 |
| Seek guidance |  | 13 | 16 |
| Seek adult help |  | 11 | 6 |
| ask questions |  | 12 | 10 |
| Expression and performance | During parent-child activities, young children's minds are active and they often associate with the teacher's words and express their thoughts to parents or teachers. | 15 | 33 |
| Expressing wishes and ideas |  | 10 | 12 |
| attracting adult attention |  | 7 | 11 |

C-P-T

| **Name** | **Description** | **Sources** | **References** |
| --- | --- | --- | --- |
| Subjective expression |  | 27 | 61 |
| Seek guidance | Young children seek help from adults when they encounter difficulties. Usually, young children tend to seek help from their parents first. Teachers will also intervene when they observe the needs of the parent-child pair. | 23 | 27 |
| Seek adult help |  | 15 | 18 |
| ask questions |  | 11 | 9 |
| Expression and performance | In parent-child activities, young children want to show off for their parents and teachers. They will attract their attention or initiate a desire to play with their parents or teachers. | 20 | 34 |
| Expressing wishes and ideas |  | 14 | 16 |
| attracting adult attention |  | 13 | 18 |
